# Supplementary material for: Mechanisms governing the pioneering and redistribution capabilities of the non-classical pioneer PU.1
Source: Nat Commun. 2020 Jan 21;11:402. doi: 10.1038/s41467-019-13960-2 (PMC6972792; doi:10.1038/s41467-019-13960-2)
Supplement: Supplementary file 7 — Source data [file 41467_2019_13960_MOESM7_ESM.zip › Source_Data/FigureS3/FigureS3J_MotifScanOutput/homerResults/motif1.similar.html]

motif1

## Information for motif1

T
A
C
G
A
T
C
G
C
A
T
G
T
C
G
A
C
G
A
T
A
C
T
G
C
G
T
A
T
A
C
G
G
C
A
T
T
G
A
C
G
C
T
A
A
G
C
T
  
Reverse Opposite:  

T
C
G
A
C
G
A
T
A
C
T
G
C
G
T
A
A
T
G
C
G
C
A
T
T
G
A
C
G
C
T
A
A
G
C
T
G
T
A
C
T
A
G
C
A
T
G
C
  

|  |  |
| --- | --- |
| p-value: | 1e-1242 |
| log p-value: | -2.862e+03 |
| Information Content per bp: | 1.561 |
| Number of Target Sequences with motif | 6002.0 |
| Percentage of Target Sequences with motif | 45.48% |
| Number of Background Sequences with motif | 4576.4 |
| Percentage of Background Sequences with motif | 12.54% |
| Average Position of motif in Targets | 337.6 +/- 289.3bp |
| Average Position of motif in Background | 318.6 +/- 203.9bp |
| Strand Bias (log2 ratio + to - strand density) | 0.0 |
| Multiplicity (# of sites on avg that occur together) | 1.30 |
| Motif File: | file (matrix) reverse opposite |

### Similar de novo motifs found

|  |  |  |  |  |  |  |  |
| --- | --- | --- | --- | --- | --- | --- | --- |
| Rank | Match Score | Redundant Motif | P-value | log P-value | % of Targets | % of Background | Motif file |
| 1 | 0.988 | A T G C C T A G T C G A G A C T C A T G G C T A A T C G C A G T G T A C G T C A A G C T | 1e-1229 | -2829.937820 | 44.99% | 12.36% | motif file (matrix) |
| 2 | 0.996 | T C G A C T A G T C A G C T A G T C G A C G A T A C T G C G T A T A C G C A G T T G A C G T C A A G C T | 1e-1215 | -2798.365046 | 42.90% | 11.18% | motif file (matrix) |
| 3 | 0.988 | A T G C C T A G T C G A G C A T A C T G G C T A T A G C G A C T G T A C T G C A | 1e-1139 | -2624.727866 | 46.16% | 14.06% | motif file (matrix) |
| 4 | 0.970 | T C G A A C G T A C T G C G T A T A G C A C G T G T A C C T G A | 1e-1115 | -2568.997292 | 48.21% | 15.74% | motif file (matrix) |
| 5 | 0.969 | A C G T A C T G C G T A A T C G A C G T A G T C C G T A A G T C G A T C | 1e-1114 | -2566.267683 | 43.08% | 12.30% | motif file (matrix) |
| 6 | 0.985 | C G A T A C T G C G T A T A G C G C A T G T A C C G T A A G C T G A T C A T G C G A T C C G A T A G C T G C T A | 1e-1100 | -2534.658835 | 44.66% | 13.48% | motif file (matrix) |
| 7 | 0.926 | A C G T A C T G C G T A A G T C A C G T G T A C C G T A | 1e-1034 | -2382.740891 | 47.45% | 16.16% | motif file (matrix) |
| 8 | 0.927 | C G A T A C T G C G T A T C A G C G A T T G A C C T G A A G C T G A T C | 1e-952 | -2192.771269 | 48.43% | 17.92% | motif file (matrix) |
| 9 | 0.938 | A C T G G T C A G A C T A C T G C G T A T A C G A C G T T G A C T G C A A C G T | 1e-935 | -2154.213353 | 35.11% | 9.21% | motif file (matrix) |
| 10 | 0.804 | C G A T A C T G G C T A C A T G A C G T T G C A G T C A A G T C | 1e-650 | -1498.344335 | 67.11% | 39.56% | motif file (matrix) |
| 11 | 0.852 | T A G C G T C A G T C A C G A T A C T G G C T A T G A C G C A T T A G C G C T A A T G C G C A T C A T G C T G A | 1e-317 | -732.163051 | 44.22% | 26.00% | motif file (matrix) |
| 12 | 0.763 | C A G T G A C T T C G A T C A G G C A T T A G C C G T A A C G T G C A T A T G C C G T A | 1e-132 | -304.435195 | 10.22% | 4.11% | motif file (matrix) |
| 13 | 0.768 | T G A C A C G T A C G T C G T A A C T G A C G T A G T C C G T A A G C T G A T C G T C A G C A T T G C A | 1e-120 | -277.409724 | 5.82% | 1.65% | motif file (matrix) |
| 14 | 0.852 | A C G T A C T G C G T A A C G T A C G T A G T C C G T A | 1e-114 | -264.711111 | 14.73% | 7.64% | motif file (matrix) |
| 15 | 0.779 | A T C G A C G T T G A C A C G T C T A G C G T A C G A T A C G T T A G C C G T A | 1e-44 | -101.600517 | 2.99% | 1.09% | motif file (matrix) |
| 16 | 0.763 | G C A T A G C T T C G A T A C G A C G T A T G C C T G A A G C T C T G A | 1e-40 | -94.021597 | 3.82% | 1.68% | motif file (matrix) |
